# Succinic semialdehyde dehydrogenase deficiency presenting with central hypothyroidism

**DOI:** 10.1002/ccr3.3504

**Published:** 2020-11-11

**Authors:** Malak Ali Alghamdi, Waleed H. Alkhamis, Dima Z. Jamjoom, Reem Al Khalifah, Nawaf Rahi Alshammari, Khalid Alsumaili, Stefan T. Arold

**Affiliations:** ^1^ Medical Genetics Division Department of Pediatrics College of Medicine King Saud University Riyadh Saudi Arabia; ^2^ Medical Genetics Division Department of Pediatrics King Saud University Medical city Riyadh Saudi Arabia; ^3^ Department of Obstetrics and Gynecology King Saud University Medical City Riyadh Saudi Arabia; ^4^ Department of Radiology and Medical Imaging College of Medicine King Saud University Riyadh Saudi Arabia; ^5^ Pediatric Endocrinology Division Department of Pediatrics College of Medicine King Saud University Riyadh Saudi Arabia; ^6^ Department of Pediatrics College of Medicine King Saud University Riyadh Saudi Arabia; ^7^ Biochemical Genetic Division Department of Pathology College of Medicine King Saud University Riyadh Saudi Arabia; ^8^ Division of Biological and Environmental Sciences and Engineering (BESE) King Abdullah University of Science and Technology (KAUST) Computational Bioscience Research Center (CBRC) Thuwal Saudi Arabia

**Keywords:** *ALDH5A1*, central hypothyroidism, developmental delay, succinic semialdehyde dehydrogenase deficiency

## Abstract

Central hypothyroidism might be another clinical sign of SSADH deficiency which prompts urinary organic acid screening for GHB in central hypothyroidism patients. Studies on GABA and thyroid hormone interaction might be a concept of a new therapy.

## Graphical abstract

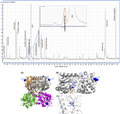

## 1 INTRODUCTION

Succinic semialdehyde dehydrogenase deficiency (SSADH) results in the accumulation of GABA which affects different neuroendocrine interactions. We report the first case with SSADH deficiency and central hypothyroidism. Upon a workup for hypotonia, a novel homozygous variant in *ALDH5A1* gene and an elevation of GABA and GHB in plasma and urine were detected.

**Figure 1:**
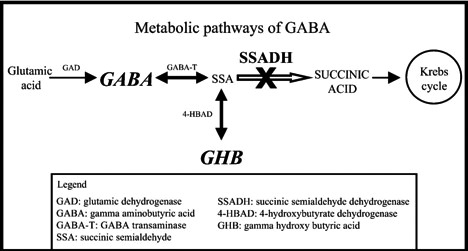
Pathway defect in SSADH deficiency and GABA, GHB, and SSA metabolites accumulation.^20^GAD; glutamic acid decarboxylase, GABA‐T; γ‐aminobutyric acid transaminase, SSADH; succinic semialdehyde dehydrogenase, GHB; γ‐Hydroxybutyric acid

Inborn errors in gamma‐aminobutyric acid (GABA) metabolism are caused by the disruption of the GABA metabolic pathway due to deficiencies in either GABA transaminase or succinic semialdehyde dehydrogenase (SSADH) (Figure 1).^1^, ^2^ SSADH deficiency (or aldehyde dehydrogenase 5A1 "ALDH5A1", gamma‐hydroxybutyric aciduria, OMIM: #271980) is a rare autosomal recessive metabolic disorder caused by a defect in the degradation pathway of gamma‐hydroxybutyric acid (GHB).^3^ SSADH deficiency leads to the accumulation of GABA and GHB in physiological fluids.^3^, ^4^ It usually presents during late infancy to early childhood as a slowly progressive disorder that impairs neurological development. The phenotype can be variable and can include cognitive impairment, hypotonia, hyporeflexia, ataxia, dystonia, myoclonus, seizures, and behavioral problems.^3^, ^5^ Brain MRI in most cases shows symmetrical hyperintensity of the globus pallidus, cerebellar dentate nuclei, and subthalamic nucleus.^6^, ^7^ A diagnosis of SSADH deficiency is established through urine Gas chromatography‐mass spectrometry (GC‐MS) organic acid analysis showing GHB ^2^ and serum gamma hydroxy butyric acid and is further confirmed by *ALDH5A1* mutation analysis.^8^

Here, we report a female infant with global developmental delay and central hypothyroidism (CH)who was diagnosed with SSADH deficiency based on high urinary excretion of GHB with a novel homozygous mutation in *ALDH5A1*gene and is a first case with SSADH deficiency presented with central hypothyroidism.

## 2 CASE REPORT

A 7‐month‐old girl who was the first child of first‐degree consanguineous Saudi parents was referred to genetic division clinic for developmental delay evaluation. She was born at term by spontaneous vaginal delivery, and her birth weight was 3 kg. The mother is a healthy 27‐year‐old primigravida, and the father is a healthy 32‐year‐old. The pregnancy, antenatal and perinatal courses were unremarkable with no reported exposure to teratogens. She was growing well until the age of 3 months, when she started to have recurrent chest infection associated with stridor, poor sucking, frequent choking, failure to thrive, hypotonia, hypoglycemia, and chronic constipation.

On examination, the child looked alert, hypoactive, and malnourished. Her length was 60 cm (<3rd percentile), her weight was 4.2 kg (<3rd percentile), and her head circumference was 40 cm (<3rd percentile). Her eye examination was normal, and a neurological examination revealed generalized hypotonia with hyporeflexia and an unremarkable systemic examination. Her developmental assessment showed an inability to sit with support, head lag, she could not turn from the supine to prone position, and she could not hold objects, but she could follow objects with both eyes.

## 3 WORKUP AND INVESTIGATIONS

She underwent extensive evaluation during an admission with pneumonia when she was 7 months old by a multidisciplinary team. The initial diagnostic work up showed normal blood gas, electrolytes, Complete Blood Count (CBC), liver profile, and renal profile. She had one episode of hypoglycemia (glucose <50 mg/dL) upon presentation to the ER. Furthermore, for hypoglycemia, she underwent a fasting challenge for 12 hr The glucose at the end was normal, and the acylcarnitine profile was within the normal range. Her hormonal profile suggested central hypothyroidism: FT4 7.23 pmol/L (normal range 12‐22 pmol/L) and TSH 2.7 mIU/L (normal range 0.25‐5 mIU/L), normal ACTH stimulation, and a normal growth hormone stimulation test (Table 1). She was started on levothyroxine 25 mcg daily.

**Table 1:** Summary of the patient's laboratory data before and after treatment with Levothyroxine

	Reference Range	8 mo old	On levothyroxine	6 wk off levothyroxine
TSH, mIU/L	0.25‐5	2.7	3.2	3.3
FT4, pmol/L	12‐22	7.23	17.1	11.4
Prolactin, mIU/L	102‐496	771	173	
IGF‐1, ng/ml	34‐172	34	99	
ACTH, pmol/L	2.2 −13.3 pmol/L	5.1	2.5	
Serum gamma hydroxy butyric acid mg/l	<1		89	50
Serum gamma amino butyric acid, μg/l	10 ‐ 20		103	
Urine gamma hydroxy butyric acid (GHB), mg/L	<5		50	25

**Figure 2:**
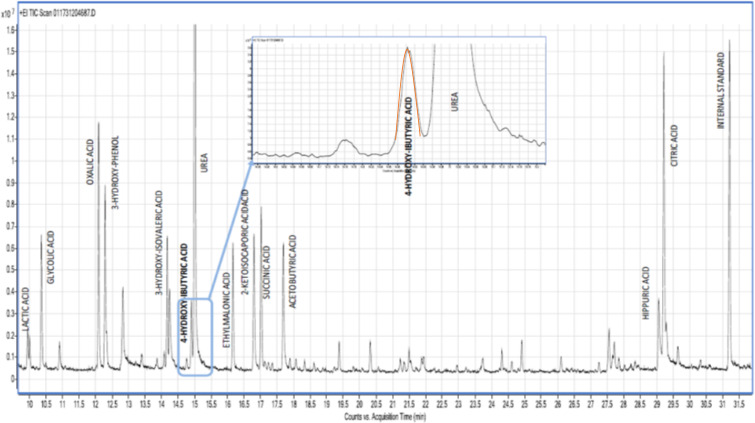
Urine organic chromatograph of this case shows a peak of 4‐hydroxybutyric acid by gas chromatography mass spectrometry

Additional investigation for potential neuro‐metabolic causes of her developmental delay showed normal homocysteine and uric acid levels. Plasma amino acid analysis revealed a slight elevation in asparagine to 115 mcmol/L (ref range 25‐91 mcmol/L) and glutamic acid to 271 mcmol/L (ref range 5‐150 mcmol/L), both of which were deemed unspecific. Urine organic acid levels were analyzed several times and showed persistently increased urinary excretion of GHB (Figure 2). Serum gamma hydroxy butyric acid levels were elevated at 89 mg/L by GC‐MS (normal < 1), serum gamma amino butyric acid levels were elevated to 103 μg/l (normal 10‐20) by Liquid chromatography‐mass spectrometry (LCMS), and urine GHB levels were elevated at 50 mg/L byGC‐MS (normal < 5 mg/L) (Table 1).

**Figure 3:**
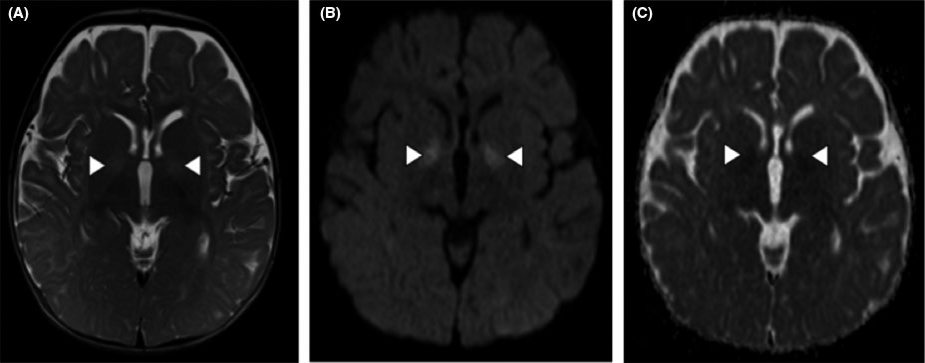
MR imaging of the brain demonstrating bilateral symmetric high signal intensity in the globi pallidi (white arrowheads) on T2‐weighted images (A), with corresponding high signal intensity of diffusion‐weighted images (DWI) (B), and low signal intensity on apparent diffusion coefficient (ADC) maps (C), consistent with diffusion restriction

**Figure 4:**
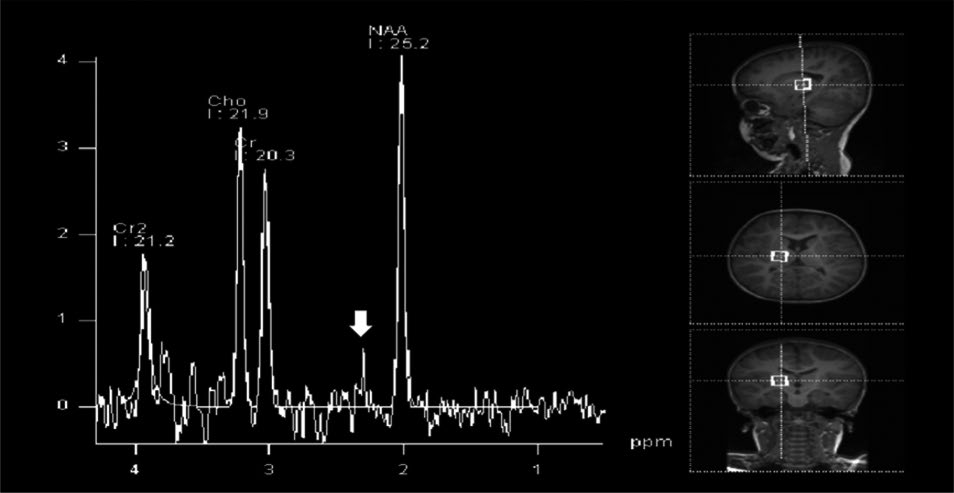
MR spectroscopy at the level of the right globus pallidus showing γ‐aminobutyric acid (GABA) and related compounds resonating at 2.3 pmp (white arrow)

Brain magnetic resonance imaging (MRI) performed at an age of 8 months revealed bilateral symmetric high signal intensity in the globus pallidus (white arrowheads) on T2‐weighted images with corresponding high signal intensity on diffusion‐weighted images (DW1) and low signal intensity on apparent diffusion coefficient (ADC) maps, consistent with diffusion restriction (Figure 3). This represents cytotoxic edema, which indicates the presence of an acute insult to this structure. At the age of 2 and a half years old, brain magnetic resonance spectroscopy (^1^H) showed a unilateral accumulation of GABA and related compounds (resonating at 2.3 ppm) at the level of the right globus pallidus (Figure 4).

Whole‐exome sequencing revealed a previously unreported homozygous variant in the *ALDH5A1* gene, NM_170740: c.209T > C; p. (Leu70Pro). Both parents were heterozygous carriers for this variant. According to the American College of Medical Genetics and Genomics criteria (ACMG), this variant is classified as a variant of uncertain significance (VUS) (class 3). Variant frequencies typically reflect our internal allele frequency of 0.00007. In silico prediction programs predicted pathogenicity in all applicable programs (SIFT, PolyPhen2, AlignGVD, MutationTaster).

Furthermore, the presence of suggestive metabolomic profiling with elevated urine and serum GABA and GHB levels, MRS peaks for GABA were considered as functional validation and predicted the pathogenicity of this variant.

## 4 CLINICAL PROGRESSION

The parents noticed improvement after the age of one year and with starting thyroid hormone replacement. She gained weight, her constipation resolved, and she progressed her developmental milestones. Currently, she is 3 years old and is able to walk and sit without support and says a few words but no sentences. Vigabatrin is a reasonable therapeutic modality to control seizures in SSADH‐deficient patients by the irreversible inhibition of GABA transaminase. Our patient has occasionally disturbed sleep patterns in the form of difficulty falling asleep, but she does not have seizure disorders, dystonia, or movement disorders. The parents have initiated the non‐pharmacologic treatments include physical therapy, occupational therapy, and speech therapy.

At the age of 3 years old, we sought to confirm the etiology of her hypothyroidism and seek further understanding of the possible link between GABA levels and the hypothalamic pituitary thyroid axis. To do so, we stopped thyroid replacement for 6 weeks and then performed thyroid‐releasing hormone (TRH) stimulation and measured GABA levels. The thyroid profile after stopping replacement showed a picture of central hypothyroidism, and TRH stimulation confirmed a tertiary form of hypothyroidism (Table 2). The mother reported no change in her child's clinical condition. The thyroid hormone replacement therapy was resumed.

**Table 2:** TRH stimulation off thyroid hormone replacement for 6 wk

	Baseline	30 min	60 min	90 min
TSH, mIU/L	3.3	28.8	20.4	37.9
FT4, pmol/L	11.4			

## 5 DISCUSSION

We describe an infant with SSADH deficiency and a unique clinical presentation of severe early onset global developmental delay, failure to thrive and evidence of a tertiary form of hypothyroidism; this is unlike the typical SSADH presentation with neurological symptoms. The diagnosis of SSADH deficiency was established based on molecular result and pathognomonic biochemical findings, including extremely high excretion of GHB and GABA in the urine and serum and by in vivo MRS‐mediated demonstration of the accumulation of GHB and GABA in the brain.

Attri et al 2016, identified clinical and genetic variables including ethnic origin among 182 patients, from 40 countries confirmed SSADH deficiency caused by *ALDH5A1* gene biallelic mutation, with the largest number of patients from the USA (24%) and (6%) from Saudi Arabia.^9^ Interestingly, the median age at diagnosis was reported to be 2 years old, and almost 80% of patients are diagnosed at 5 years old.^9^ However, our patient started showing manifestations at the age of 3 months, and this is considered to be earlier than other reported cases.^9^ Of all published cases worldwide, only 10% of cases have been diagnosed after 10 years, and few cases have been diagnosed during adult life.^12^ Epilepsy is found in half of the cases.^8^, ^10^, ^11^ Adult presentations of SSADH deficiency further include neuropsychiatric disorders, such as obsessive‐compulsive disorder (OCD), anxiety, seizure, sleep disturbance, and dementia.^12^, ^13^ The pathophysiologic mechanisms underlying the heterogeneous clinical presentations of SSADH deficiency are still under investigation. Given what we currently know, a relationship between the level of GHB accumulation in physiological fluids and the severity of the disease seems unlikely.^8^

One explanation for our patient's unusual presentation is the coexistence of tertiary hypothyroidism. Tertiary hypothyroidism is a condition caused by insufficient TRH stimulation of an otherwise normal pituitary and thyroid gland. Central hypothyroidism is approximately 1000‐fold rarer than primary hypothyroidism, and it may present as an isolated finding or may be associated with other pituitary hormone deficiencies. Central hypothyroidism could be secondary to genetic defects, such as those in *TRHR*, *POU1F1*, *PROP1*, *HESX1*, NKX2E, THRA, *SOX3*, *LHX3*, *LHX4,* and *TSHB*, which encode hypothalamic and pituitary transcription factors; the thyroid‐stimulating hormone(TSH) beta subunit; or the TRH receptor. Some of these genetic defects result in isolated CH or combined pituitary hormone deficiency with variable phenotypical spectra similar to those of primary hypothyroidism, including fatigue, constipation, dry skin, and weight gain, and might present early in the neonatal period as a severe phenotype including jaundice, macroglossia, coarse cry, failure to thrive, retarded growth, umbilical hernia, and hypotonia. Moreover, the presence of signs and symptoms of other pituitary hormone deficiencies, such as hypoglycemia, adrenal insufficiency, and midline defect, may mask underlying CH.^14^

Several of our patient's signs and symptoms can be explained by hypothyroidism; these include hypotonia, retarded growth, and constipation. She was diagnosed with tertiary isolated hypothyroidism at the age of 7 months old with no other hormonal involvement and no midline defect or craniofacial anomalies as well as a normal pituitary on MRI. Whole‐exome sequencing did not identify any genetic mutation in a gene known to be associated with central hypothyroidism.

**Figure 5:**
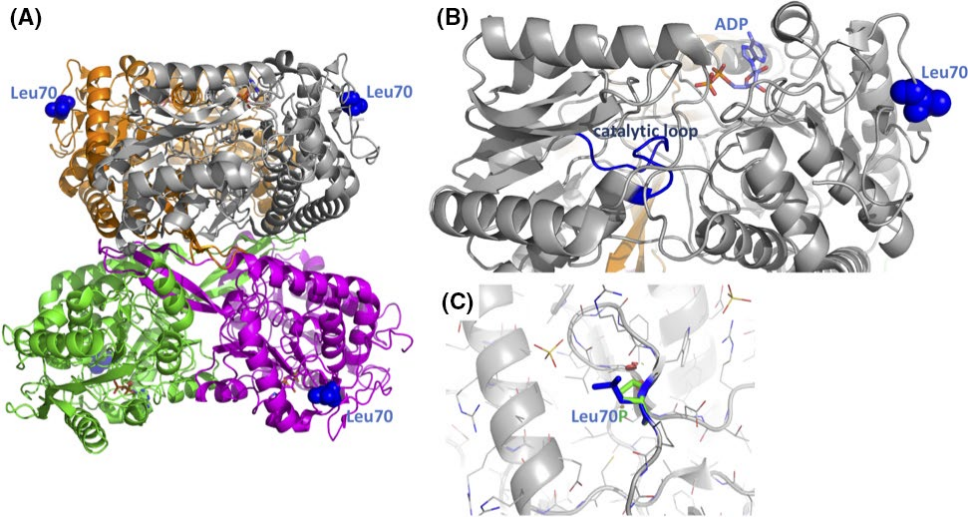
Molecular effect of Leu70Pro. A, Structure of the tetrameric*ALDH5A1*(PDB accession 2W8R). The four monomers are color coded. Leu70 is shown as blue sphere model. B, Leu70 (blue spheres) is distal to the active site (ADP and the catalytic loop are highlighted). C, close‐up view on Leu70 (blue stick model) and the in silico‐substituted Pro (green)

To the best of our knowledge, we have not been able to identify any of the known causes of central hypothyroidism in our patient, but there are a few reasons why we believe that the tertiary hypothyroidism observed in our patient might be directly associated with SSADH deficiency. Various mechanisms aimed at explaining the interaction between GABA and the thyroid hormone axis have been suggested based on animal and human studies. GABA exerts negative feedback on thyroid hormone homeostasis by inhibiting thyroid synthesis via the inhibition of TRH and TSH.^15^ Thyroid hormones exert different effects on developed and developing brains. They stimulate GABA function in the developing brain and inhibit GABA function in the adult brain (Figure 5).^15^

Hypothyroidism has not been described as a feature of SSADH deficiency in the 450 patients described in the literature thus far. However, given the involvement of GABA in the regulation of central thyroid homeostasis, it might be worth systematically screening patients with SSADH deficiency for hypothyroidism. This has important implications, especially for brain development.

Our patient has a homozygous novel *ALDH5A1* variant. Although we have not been able to perform in vitro studies (eg, variant overexpression and wild type rescue) to confirm the pathogenicity of these variants, the presence of the biochemical phenotype demonstrated by high urinary and brain concentrations for GHB and GABA, respectively, is a convincing argument in favor of the variant's pathogenicity. Furthermore, the MRI findings, including deep gray matter involvement (Figure 3), are deemed characteristic of SSADH deficiency.

The three‐dimensional structure of the tetrameric ALDH5A1 protein has been determined [PDB accession nr 2W8O, ^16^]: Leu70 is located in a surface loop region, more than 15 Å away from the active site, ruling out a direct effect on substrate binding and catalysis (Figure 5). However, the substitution of Leu70 by a smaller and conformationally restricted proline is predicted to destabilize the structure locally. This destabilization might affect the overall enzyme stability and longevity, and/or allosterically affect the dynamics of substrate catalysis which is further confirm the pathogenicity of the variant.

Wiens and Trudeau (2006) reviewed the effect of thyroid hormone on GABA production and degradation. The data showed that hypothyroidism decreased glutamic dehydrogenase (GAD) activity and subsequently GABA levels in the developing brain and either had no effect on the adult brain or increased enzymatic activity and GABA levels. Additionally, it has been shown that thyroid hormone supplementation rescues GAD activity in the developing brain. In our patient, we observed high levels of serum and urinary GABA on thyroid hormone supplementation, and these were reduced after two months of thyroid hormone supplementation. However, the levels were still higher than those in the control group due to the genetic defect in the SSADH gene.^15^

There are currently several treatment options for patients with SSADH deficiency. Vigabatrin is known to decrease GHB concentrations in CSF^17^ and is considered one of the therapeutic modalities in SSADH deficiency. Other therapeutic modalities were reviewed, such as ketogenic diet, nutrient supplementation(such as taurine),^18^ GABA receptor antagonist, and gene therapy.^19^ The rest of the treatments are symptomatic and directed toward the management of seizures and neurobehavioral disturbances.

In conclusion, SSADH deficiency is a rare IEM that is characterized by progressive neurological disorder that results in cognitive impairment, hypotonia, hyporeflexia, ataxia, dystonia, myoclonus, seizures, and behavioral problems. At the early stage of clinical presentation, the condition is difficult to differentiate from static encephalopathy, and biochemical phenotyping employing the analysis of urinary organic acids should therefore be performed in all patients at risk to allow early diagnosis. As suggested by this case report, tertiary hypothyroidism might be another clinical sign to prompt urinary organic acid analysis.

## INFORMED CONSENT

Informed consent was obtained from the parents.

## CONFLICT OF INTEREST

None declared.

## AUTHOR CONTRIBUTIONS

Attached document.

## Data Availability

Data available within the article or its supplementary materials.
